# Production and Application of a Soluble Hydrogenase from *Pyrococcus furiosus*

**DOI:** 10.1155/2015/912582

**Published:** 2015-10-12

**Authors:** Chang-Hao Wu, Patrick M. McTernan, Mary E. Walter, Michael W. W. Adams

**Affiliations:** Department of Biochemistry and Molecular Biology, University of Georgia, Athens, GA 30602, USA

## Abstract

Hydrogen gas is a potential renewable alternative energy carrier that could be used in the future to help supplement humanity's growing energy needs. Unfortunately, current industrial methods for hydrogen production are expensive or environmentally unfriendly. In recent years research has focused on biological mechanisms for hydrogen production and specifically on hydrogenases, the enzyme responsible for catalyzing the reduction of protons to generate hydrogen. In particular, a better understanding of this enzyme might allow us to generate hydrogen that does not use expensive metals, such as platinum, as catalysts. The soluble hydrogenase I (SHI) from the hyperthermophile *Pyrococcus furiosus*, a member of the euryarchaeota, has been studied extensively and used in various biotechnological applications. This review summarizes the strategies used in engineering and characterizing three different forms of SHI and the properties of the recombinant enzymes. SHI has also been used in *in vitro* systems for hydrogen production and NADPH generation and these systems are also discussed.

## 1. Introduction

Hydrogen is a potential renewable and carbon neutral energy carrier. It has three times the energy content per unit mass of fossil fuels [1]. The concept of replacing current gasoline-based vehicles with hydrogen fuel cell vehicles (HFCVs) has gained a lot of attention recently [2]. A major advantage of HFCVs is that water is the only waste product, and hence they eliminate the harmful exhaust of current vehicles, thereby benefiting human health and the climate [2, 3]. With the introduction of commercially available HFCVs in many counties in 2015, the demand for hydrogen is anticipated to dramatically increase in the near future [3]. Unfortunately, current methods of producing hydrogen rely on fossil fuels and are expensive. They include steam reforming of natural gas, which produces greenhouse gases, and electrolysis to split water uses the expensive metal platinum as a catalyst [4]. New and renewable methods are obviously needed for the generation of hydrogen and biological-based systems have a great deal of potential.

The enzyme hydrogenase catalyzes the simplest chemical reaction in nature, the reversible interconversion of protons, electrons, and hydrogen gas: 2H^+^ + 2e^−^↔H_2_. Such enzymes are widespread in bacteria and Archaea and are even found in some Eukarya [5]. Hydrogenases enable organisms to remove excess reducing power generated during metabolism by evolving hydrogen, or they can oxidize hydrogen to generate reducing power for growth [6]. Hydrogenases can be classified into three types based on the metal content of their catalytic sites, and they are referred to as [NiFe] hydrogenases, [FeFe] hydrogenases, and mononuclear Fe hydrogenases [7]. The [NiFe] hydrogenases are the most ubiquitous and have been extensively studied [5]. They are further classified into four different types (groups 1–4) based on the peptide sequence used to bind the [NiFe]-containing active site [7]. Group 1 [NiFe] hydrogenases are the best studied among the four groups [5]. The assembly of the [NiFe] catalytic site of these hydrogenases requires eight maturation proteins, based on the mechanism elucidated for* Escherichia coli* hydrogenase 3 [8]. The [NiFe] hydrogenases are also reversibly inactivated in the presence of oxygen [9].

Herein we focus on the [NiFe] hydrogenases of* Pyrococcus furiosus*, a strictly anaerobic archaeon that grows optimally at 100°C. This organism utilizes carbohydrates as a carbon source for growth and generates acetate, carbon dioxide, and hydrogen gas as end products.* P. furiosus* contains three different types of [NiFe] hydrogenase, a membrane-bound enzyme (MBH) and two soluble hydrogenases (SHI and SHII). MBH is the hydrogenase responsible for producing H_2_ during its fermentative metabolism wherein it oxidizes the reduced ferredoxin generated during the oxidation of glucose to acetate [15, 16]. In contrast, SHI and SHII utilize NADP(H) and NAD(H) as electron carriers, respectively, and while their functions have not been established, it is assumed that they can recycle some of the H_2_ produced by MBH under the appropriate growth conditions. All three hydrogenases have been purified and characterized [6, 17–19]. This review focuses on the engineering, properties, and applications of SHI.

## 2. Expression and Purification

**Figure 1:**
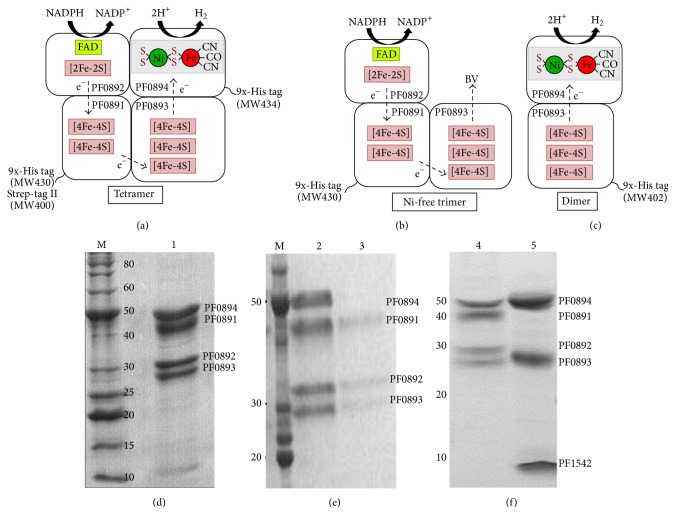
Models of tetrameric (a), Ni-free trimeric (b), and dimeric (c) forms of SHI. These are modified from [10] and are based on the cofactor and iron-sulfur cluster contents in sequence analysis. The proposed electron flow from NADPH oxidation to hydrogen evolution is also shown. Four different strains of* P. furiosus* were constructed to obtain the various forms of SHI. They are designated as MW400, MW430, MW434, and MW402 and their properties are listed in Table 2. These were used to prepare PF0891 Strep-tag II SHI [11], PF0891 9x-His tag SHI [12], PF0894 9x-His tag SHI [12], and PF0893 9x-His tag dimeric SHI [13], respectively. SDS PAGE gels show the purity of the different forms: (d) lane 1, Strep-tag II tetrameric SHI; (e) lane 2, 9x-His tag tetrameric SHI; lane 3, 9x-His tag Ni-free trimeric SHI; (f) lane 4, native SHI; lane 5, 9x-His tag dimeric SHI (PF1542 is an unrelated protein that is a persistent contaminant that copurified with dimeric SHI). The SDS PAGE gel data were modified from [11–13].

*P. furiosus* SHI is a heterotetramer encoded by a four-gene operon (PF0891–0894). A structural model of SHI has been proposed based on sequence analyses of the four subunits [10]. As shown in Figure 1, PF0894 is the subunit harboring the Fe- and Ni-containing catalytic site wherein the Fe atom has three diatomic ligands, one -CO and two -CN. PF0892 contains the flavin and a [2] cluster and is the site of interaction with NADP(H). PF0891 and PF0893 contain two and three [4] clusters, respectively, for electron transfer between the flavin and the active site. SHI was first purified and characterized using four chromatographic steps, which yielded the intact heterotetramer [6]. The yield from this purification was very low and an improvement in yield was needed in order to generate the enzyme for detailed characterization studies.

In order to improve the yield of SHI, an attempt was made to heterologously express SHI in* E. coli*, with coexpression of the genes encoding the accessory proteins that are necessary for proper assembly of the [NiFe] active site [10]. This was also the first example of heterologously expressing a functional [NiFe] hydrogenase in* E. coli*, as well as demonstrating expression of a hydrogenase in a distantly related host. Unfortunately, the yield of this heterologous expressed SHI was lower than the natively purified SHI from* P. furiosus* [10]. Although the expression of SHI in a genetically tractable host, such as* E. coli*, was a significant achievement, this system was not suitable to produce large amounts of this enzyme.

Once a genetic system was established in* P. furiosus*, the host organism could be used to both overproduce and engineer SHI [20]. The genetic system was established by removing the* pyrF* gene in a genetically tractable strain of* P. furiosus* termed COM1.* pyrF* is essential for uracil biosynthesis and allows for selection and counter selection based on uracil biosynthesis in a minimal medium. Moreover, it was demonstrated that the genes encoding SHI could be deleted from* P. furiosus* without any apparent effect on cell growth [20]. This suggested that SHI was not an essential enzyme and could be engineered in various ways without affecting the metabolism of* P. furiosus*, and this proved to be the case.

**Figure 2:**
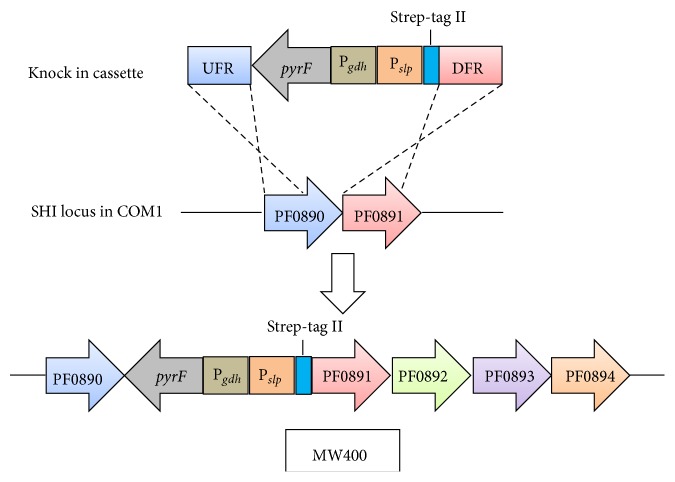
Genetic strategy for overexpression of SHI. MW400 is used as the example. The knock in cassette contains upstream flanking region (UFR) and downstream flanking region (DFR) homologous to PF0890 and PF0891, respectively. This cassette also contains a selectable marker* pyrF* with the promoter P_*gdh*_, the promoter for the S-layer protein (P_*slp*_) to drive expression of the SHI genes, and a Strep-tag II at the N-terminus of PF0891. By homologous recombination, this cassette was inserted into the SHI locus in* P. furiosus* COM1.

In the first attempt to overexpress the four genes encoding SHI and to affinity-tag the enzyme [11], transcription of SHI was put under the control of a strong constitutive promoter, P_*slp*_, which controls expression of the gene encoding the S-layer protein. In addition, PF0891 was engineered to include a Strep-II affinity tag (Figure 2). A Strep tag was chosen instead of a polyhistidine tag as the latter might interfere with the incorporation of nickel into the catalytic site of SHI. Using this approach and a one-step affinity purification, approximately seven times more SHI (per gram of cells) was purified from the cytoplasmic fraction of* P. furiosus* compared to the original purification of SHI [11]. Interestingly, expression of the genes encoding the [NiFe]-maturation proteins was at the same level in the recombinant strain as in the parent strain even though the genes encoding SHI (under the control of P_*slp*_) were increased by about 10-fold. The native levels of the maturation proteins were therefore able to synthesize almost an order of magnitude more SHI and produce the active enzyme.

Since a functional SHI is not required for growth of* P. furiosus* [20], this allowed the engineering of nonfunctional forms that did not utilize H_2_ and/or NADP(H) as substrates. A dimeric version of SHI that contained only PF0893 and PF0894 was successfully produced (Figure 1). This enzyme evolved H_2_ from artificial electron donors but did not oxidize NADPH, as it lacked the NADPH-oxidizing subunit [13]. Engineering dimeric SHI also involved the development of another selectable marker, arginine decarboxylase (*pdaD*), to be used for genetic manipulations in* P. furiosus*. In addition, the purified dimeric SHI had a polyhistidine (9-His) tag at the N-terminus of PF0893. The results demonstrated that this type of tag does not interfere with the assembly of nickel into the catalytic site of SHI as the enzyme retained its H_2_-production activity (using an artificial dye as the electron donor) after purification using the immobilized nickel-affinity chromatography step.

Based on the success in engineering and purifying 9x-His tag dimeric SHI, the tetrameric SHI was engineered to contain a polyhistidine affinity tag to determine if this would improve the efficiency of purification compared to the Strep-tag II tetramer [12]. The same strategy to overexpress SHI (tagging at the N-terminus of PF0891) was used, except that the Strep-tag II was replaced by a 9x-His tag (Figure 2). This resulted in an 8-fold improvement in the yield compared to the Strep-tag II and a 50-fold higher yield of SHI compared to the original native purification. A comparison of the yields for the different purification procedures is shown in Table 1 and the strains constructed for SHI expression are shown in Table 2.

During the affinity purification of the His-tagged tetrameric form of SHI, a trimeric form was observed eluting from the affinity column that lacked the large [NiFe]-containing subunit (PF0894, see Figure 1). This Ni-free trimeric SHI represented approximately 2% of total SHI [12]. This discovery supports the proposed maturation mechanism of [NiFe] hydrogenases in which the three subunits of the* P. furiosus* enzyme (PF0891–PF0893) form a trimeric complex before the catalytic subunit (PF0894) is assembled [8]. This complex then binds to the catalytic subunit to generate the active enzyme. Hence, in an overexpressed strain, the processing machinery may not be able to keep up with the production of the four protein subunits, such that there is a very slight excess of the Ni-free trimeric form. These results are also consistent with the notion that the catalytic subunit alone cannot be expressed and isolated in an active form and needs the other hydrogenase subunits to be processed. For example, when the catalytic subunit of a cytoplasmic hydrogenase of* Thermococcus kodakarensis* was expressed in* E. coli*, the purified subunit was inactive and contained Fe, Zn, and Ca atoms but not Ni, indicating that it was not properly assembled [21].

## 3. Properties

SHI is classified as a group 3 bidirectional [NiFe] hydrogenase based on the amino acid sequence that surround the four cysteinyl residues that coordinate the [NiFe] catalytic site [7]. In* in vitro* assays, SHI oxidizes H_2_ and reduces NADP^+^ and can also reduce protons* in vitro* to evolve H_2_ using NADPH as the electron donor [22]. Kinetic studies on SHI showed that it has ten times higher hydrogen consumption activity than hydrogen production and has a high affinity for hydrogen (*K*_*m*_ 20 *μ*M) and NADP^+^ (*K*_*m*_ 37 *μ*M) [6, 23, 24]. This suggested that the physiological function of SHI is to regenerate NADPH from the hydrogen produced by MBH [24]. However, since the SHI deletion mutant strain did not have a phenotype under the growth conditions used in the laboratory, the true physiological function of SHI is still a mystery [20]. As shown in Figure 1, it is predicted that NADPH binds to the flavin-containing subunit PF0892 and the electrons are transferred through the iron-sulfur clusters of PF0892, PF0891, and PF0893 and finally to the catalytic site in PF0894 in order to evolve hydrogen. As expected, the dimeric form of SHI did not interact with NADP(H) but, interestingly, it accepted electrons directly from pyruvate ferredoxin oxidoreductase (POR) to produce hydrogen in the absence of an intermediate electron carrier. This two-enzyme system therefore directly oxidized pyruvate to hydrogen gas (and acetyl-CoA) [13]. Potentially, the POR subunit that would normally reduce ferredoxin is able to directly transfer electrons to iron-sulfur clusters in dimeric SHI that are exposed due to the lack of the other two subunits (Figure 1).

*P. furiosus* is a hyperthermophile that grows at 100°C so it would be expected that SHI is extremely stable at high temperature and this proved to be the case. The half-life of SHI at 90°C (as measured by its hydrogen evolution activity) is 14 hours for the native enzyme and 6 hours for the affinity tagged enzyme [12]. SHI is not a very oxygen-sensitive enzyme. The half-life in air at 25°C (as measured by loss of hydrogen evolution activity at 80°C) was 21 hours for native SHI and 25 hours for the affinity tagged version [11]. SHI is much less sensitive to inactivation by oxygen compared to the well-characterized group 1 [NiFe] hydrogenases, which are typically inactivated within an hour after exposure to oxygen [25]. Although it is regarded as a strictly anaerobic microorganism,* P. furiosus* is also resistant to oxygen and contains a mechanism of oxygen detoxification. This allows it to grow even in the presence of 8% (v/v) oxygen. SHI does not contribute to the resistance mechanism as the SHI deletion strain behaved similarly to the parent strain [26]. The general resistance to oxygen of SHI is an attractive property for biofuel-related applications.

SHI has been characterized previously by electron paramagnetic resonance (EPR) and Fourier transform infrared (FTIR) spectroscopy [6, 27–29]. The EPR properties of the enzyme are consistent with the iron-sulfur clusters predicted in the model as shown in Figure 1 [6, 29]. EPR can also be used to follow the electronic state of the [NiFe] active site. In general, the Ni atom in [NiFe] hydrogenases typically exhibits three paramagnetic states referred to as Ni-A, Ni-B, and Ni-C. Ni-A is referred to as the inactive unready state and requires incubation under reducing conditions for hours to become active. Ni-B is referred to as the inactive ready state of the enzyme and this can be reactivated within minutes under reducing conditions [5]. These EPR-active states are further distinguished by the type of oxygen ligand bound in the active site. Ni-A is believed to harbor a peroxide ligand while Ni-B is thought to harbor a bound hydroxide ligand, which may explain the faster reactivation for Ni-B state as the hydroxide would be easier to remove upon reduction. Ni-C represents the active ready state of the enzyme, which is free of any oxygen species, and performs the catalytic reaction with hydrogen [5]. These EPR detectable states (Ni-A-like, Ni-B-like, Ni-C) have been observed within* P. furiosus* SHI although a heat treatment step was required to observe some of these signals [27].

The diamagnetic states of the Ni atom in [NiFe] hydrogenases can be observed by FTIR, which detects the vibration frequencies of the CO and CN ligands bound to the Fe atom in the active site. The FTIR signals on the CN ligand of SHI have been reported [28]. The frequencies at 1959, 1950, 1967, and 1954 cm^−1^ were assigned to the Ni_u/r_-A/B, Ni_a_-S, Ni_a_-C, and Ni_a_-SR states, respectively [5, 28]. These are in agreement with the data obtained from the extensively studied group 1 hydrogenases. The results from X-ray absorption spectroscopy also show that the nickel coordination geometry of SHI is identical to that of the active site of the group 1 hydrogenases [30]. Taken together, all of these data show that the [NiFe] site of SHI assumes the same redox states and similar architecture as the catalytic sites of the standard group 1 hydrogenases. Indeed, SHI was recently used as a model [NiFe] hydrogenase to investigate the catalytic mechanism using nanosecond transient infrared and visible absorbance spectroscopy [31]. This approach identified three new catalytic intermediates and established the first elementary mechanistic description of catalysis by a [NiFe] hydrogenase.

## 4. Biotechnological Applications

Like all hydrogenases, SHI catalyzes the reversible oxidation of hydrogen but it is extremely unusual in that it is one of the few hydrogenases that use NAD(P)H as an electron carrier. Hence, SHI can oxidize NADPH to produce hydrogen or can use hydrogen to reduce NADP^+^, and applications exist that rely on both of these reactions. In general industrial applications, oxidoreductase-type enzymes have been used as biocatalysts in organic synthesis where they catalyze stereoselective reductions. The products from these syntheses include pharmaceuticals, artificial flavors, and agrochemicals [32]. However, such oxidoreductases require a cofactor, either NADH or NADPH, as a source of reductant, but these are too expensive to be used directly in industrial synthesis. SHI was the first hydrogenase reported to be used in an application to regenerate NADPH using hydrogen as the source of reducing power. It was used to regenerate NADPH in enantioselective reductive reactions* in vitro* catalyzed by* Thermoanaerobium* alcohol dehydrogenase [32]. With SHI and hydrogen, the yield of product was greatly improved compared to using NADPH alone. It was also reported that SHI was able to produce approximately 200 *μ*mol of NADPH in 100 hours of repetitive batch reactions. SHI was used at 40°C, at which temperature it had a half-life of 208 hours [32]. These results demonstrate that SHI functions efficiently at temperatures far below its optimum (>90°C). Instead of batch reactions, SHI has been attached on graphite and glass beads in a continuous NADPH production system, and it was also demonstrated that SHI was electrochemically active in the presence of hydrogen in cyclic voltammetric experiments [33]. Electrochemical and spectrophotometric studies also supported the potential applications on biofuel cell and bioelectrocatalytic applications, where SHI immobilized electrodes are used to replace electrocatalysts, such as platinum [34, 35].

SHI has also been used in several different hydrogen production systems. In an* in vitro* hydrogen photoproduction system, SHI accepted electrons from a light activated semiconductor, titanium dioxide (TiO_2_) [36, 37]. In this system, a mediator, methyl viologen, was used initially as the electron carrier, but it was found that SHI accepted electrons directly from the photoactivated TiO_2_. Improvements on this system have also been reported [38, 39]. Instead of using a mixture of SHI and TiO_2_, a two-compartment system was developed that was separated by a membrane. Anodized tubular TiO_2_ electrode (ATTE) was placed at the anode and SHI immobilized on the ATTE was used at the cathode. ATTE on the anode splits water into protons and oxygen, and the electrons were conducted into a solar cell and then transferred to cathodic SHI immobilized ATTE for hydrogen production. This system avoided using the rate-limiting step of electron transfer from the photocatalyst to the enzyme thereby improving system performance [38]. It was also shown that SHI and ATTE could be chemically cross-linked, which had a higher hydrogen production yield compared to either a slurry of SHI-ATTE or the direct adsorption of SHI onto ATTE [39].

**Figure 3:**
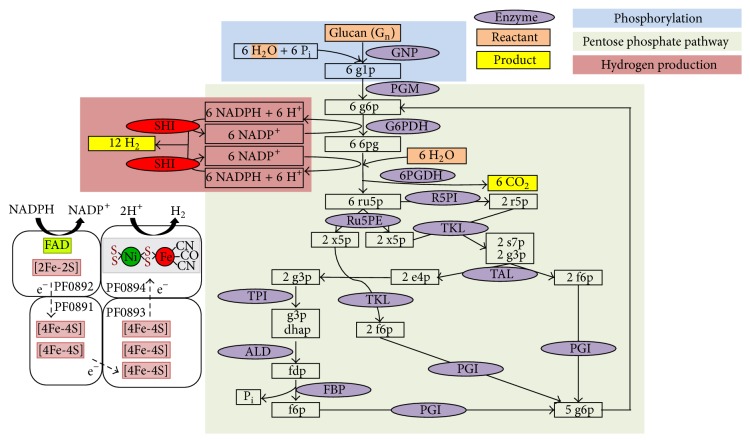
Biohydrogen production from glucan and water via SyPaB. SHI is colored in red and the model of tetrameric SHI (see Figure 1) shows how NADPH is oxidized to produce hydrogen. The abbreviations are GNP, glucan phosphorylase; PGM, phosphoglucomutase; G6PDH, G-6-P dehydrogenase; 6PGDH, 6-phosphogluconate dehydrogenase; R5PI, phosphoribose isomerase; Ru5PE, ribulose 5-phosphate epimerase; TKL, transketolase; TAL, transaldolase; TPI, triose phosphate isomerase; ALD, aldolase; FBP, fructose-1,6-bisphosphatase; PGI, phosphoglucose isomerase. g1p, glucose-1-phosphate; g6p, glucose-6-phosphate; 6pg, 6-phosphogluconate; ru5p, ribulose-5-phosphate; x5p, xylulose-5-phosphate; r5p, ribose-5-phosphate; s7p, sedoheptulose-7-phosphate; g3p, glyceraldehyde-3-phosphate; e4p, erythrose-4-phosphate; dhap, dihydroxyacetone phosphate; fdp, fructose-1,6-diphosphate; f6p, fructose-6-phosphate; and Pi, inorganic phosphate. Modified from [14].

SHI is also essential for a hydrogen-producing* in vitro* system that has been developed whereby a variety of sugars are completely oxidized to hydrogen and carbon dioxide. This stemmed from an initial report showing that purified SHI could successfully be used in an* in vitro* hydrogen production system when combined with purified glucose dehydrogenase (GDH) from* Thermoplasma acidophilum* [40]. In this system, glucose was oxidized by GDH to generate NADPH and the NADPH was utilized by SHI for hydrogen production. NADP^+^ produced by SHI was recycled by GDH for continual hydrogen production. In addition, this two-enzyme hydrogen production system also generated a high value by-product, gluconic acid, from glucose oxidation [40]. This system was further modified to use renewable resources for hydrogen production, including sucrose, lactose, cellulose, xylan, starch, and pretreated aspen wood, where corresponding enzymes were added to produce monosaccharides. These monosaccharides were further hydrolyzed by the appropriate enzymes for the hydrogen production [41, 42]. This has now been developed into a novel and highly efficient method for* in vitro* hydrogen production using a range of sugars and various mixtures of purified enzymes where, in all cases, SHI is the catalyst for hydrogen production [43]. Compared to biological fermentations (4 H_2_/glucose), this cell-free synthetic pathway biotransformation (SyPaB) has a three-time higher theoretical yield (12 H_2_/glucose). Different sugar sources can be used, including starch [43], cellulosic materials [44], xylose [45], and sucrose [46]. SyPaB has three basic modules for biohydrogen production as shown in Figure 3, for example, using starch as the sugar source, which is first phosphorylated using inorganic phosphate rather than ATP to produce glucose 6-phosphate (G6P). G6P is oxidized by the pentose phosphate pathway in the second module in order to generate NADPH and to regenerate G6P. In the third module, NADPH produced in the second module is used for hydrogen generation by SHI [43]. This coupled system of SHI and pentose phosphate pathway for biohydrogen production was first described by Woodward et al. [47]. The net reaction is given by the following equation: C_6_H_10_O_5_ (l) + 7 H_2_O (l) → 12 H_2_ (g) + 6 CO_2_ (g). The rate of hydrogen production from glucose was reported to be two times higher than the other substrate sources [48]. Moreover, this pathway exhibited one of the highest biohydrogen generation rates, 157 mmol/L/h, while using glucose 6-phosphate as the electron source [48]. The cost of biohydrogen production by SyPaB using carbohydrates as the source of reductant could be 40–75% lower than commodity prices [14]. Interestingly, a very recent study showed that glucose and xylose from plant biomass could be completely converted to hydrogen by the* in vitro* enzymatic pathway containing* P. furiosus* SHI [49], which bodes well for the future development of this synthetic approach.

## 5. Conclusions

*P. furiosus* SHI has been successfully overexpressed in the native host in affinity-tagged forms and can be purified from the cytoplasmic extract in high yield by a single chromatography step. The most efficient purification used a polyhistidine tag, and the overall yield was 50 times higher than that obtained in the original purification of native SHI, which used multistep column chromatography. SHI has been characterized spectroscopically using EPR and FTIR. Although SHI is classified as a group 3 hydrogenase, the properties of its [NiFe] catalytic site appear to be very similar to those of the extensively characterized group 1 enzymes. SHI has a wide temperature spectrum of enzyme activity (30–95°C) and is less sensitive to oxygen inactivation than typical [NiFe] hydrogenases. It is one of the few hydrogenases that uses NADP(H) as an electron carrier and this has been taken advantage of in several biotechnological applications. These include using SHI to regenerate NADPH with hydrogen as the electron donor for NADPH-dependent oxidoreductase reactions and using SHI to produce hydrogen from NADPH in cell-free synthetic pathways that oxidize a variety of sugars, as well as those in plant biomass, completely to hydrogen gas and carbon dioxide. The availability of significant quantities of recombinant and affinity-tagged SHI should facilitate the further development of these applications, as well as enabling more fundamental structure-function studies of this fascinating enzyme.

## Figures and Tables

**Table 1:** Yields of SHI from different expression systems.

Protein	Expression host	Affinity tag	Purification steps	Protein yield (mg)^1^	Reference
Native SHI	*P*. *furiosus*	—	4	2.5	[6]
Recombinant SHI	*E*. *coli*	—	3	0.16	[10]
Strep-tag II SHI	*P*. *furiosus*	Strep-tag II	1	17	[11]
9x-His Dimeric SHI	*P*. *furiosus*	9x-His tag	2	16	[13]
9x-His SHI	*P*. *furiosus*	9x-His tag	1	135	[12]

^1^Protein yield from 100 g of cells (wet weight).

**Table 2:** Strains for SHI expression.

Strain designation	Genotype	Deleted or inserted ORF/elements	Reference
MW400	P_*slp*_ Strep-tag II-*shIβγδα*	P_*slp*_ Strep-tag II, P_*gdh*_ *-pyrF*	[11]
MW402	Δ*shIβγδα* P_*slp*_ 9x-His-*shIδα*	P_*slp*_ 9x-His-*shIδα*, P_*pdaD*_-*pdaD*	[13]
MW430	P_*slp*_ 9x-His-*shIβγδα*	P_*slp*_9x-His, P_*gdh*_ *-pyrF*	[12]
MW434	P_*slp*_ 9x-His-*shIβγδα*	P_*slp*_9x-His, P_*gdh*_ *-pyrF*	[12]
